# Mitral valve regurgitation assessed by intraventricular CMR 4D-flow: a systematic review on the technological aspects and potential clinical applications

**DOI:** 10.1007/s10554-023-02893-z

**Published:** 2023-06-16

**Authors:** Yasaman Safarkhanlo, Bernd Jung, Benedikt Bernhard, Eva S. Peper, Raymond Y. Kwong, Jessica A. M. Bastiaansen, Christoph Gräni

**Affiliations:** 1 Department of Cardiology, Inselspital, Bern University Hospital, University of Bern, Freiburgstrasse 10, 3010 Bern, Switzerland; 2 Department of Diagnostic, Interventional and Pediatric Radiology, Inselspital, University Hospital Bern, University of Bern, Bern, Switzerland; 3 Noninvasive Cardiovascular Imaging Section, Cardiovascular Division, Department of Medicine, Brigham and Women’s Hospital, Harvard Medical School, Boston, MA USA; 4 Translation Imaging Center (TIC), Swiss Institute for Translational and Entrepreneurial Medicine, Bern, Switzerland

**Keywords:** 4D-flow, Mitral valve regurgitation, Cardiac magnetic resonance imaging

## Abstract

Cardiac magnetic resonance (CMR) four-dimensional (4D) flow is a novel method for flow quantification potentially helpful in management of mitral valve regurgitation (MVR). In this systematic review, we aimed to depict the clinical role of intraventricular 4D-flow in MVR. The reproducibility, technical aspects, and comparison against conventional techniques were evaluated. Published studies on SCOPUS, MEDLINE, and EMBASE were included using search terms on 4D-flow CMR in MVR. Out of 420 screened articles, 18 studies fulfilled our inclusion criteria. All studies (n = 18, 100%) assessed MVR using 4D-flow intraventricular annular inflow (4D-flow_AIM_) method, which calculates the regurgitation by subtracting the aortic forward flow from the mitral forward flow. Thereof, 4D-flow jet quantification (4D-flow_jet_) was assessed in 5 (28%), standard 2D phase-contrast (2D-PC) flow imaging in 8 (44%) and the volumetric method (the deviation of left ventricle stroke volume and right ventricular stroke volume) in 2 (11%) studies. Inter-method correlations among the 4 MVR quantification methods were heterogeneous across studies, ranging from moderate to excellent correlations. Two studies compared 4D-flow_AIM_ to echocardiography with moderate correlation. In 12 (63%) studies the reproducibility of 4D-flow techniques in quantifying MVR was studied. Thereof, 9 (75%) studies investigated the reproducibility of the 4D-flow_AIM_ method and the majority (n = 7, 78%) reported good to excellent intra- and inter-reader reproducibility. Intraventricular 4D-flow_AIM_ provides high reproducibility with heterogeneous correlations to conventional quantification methods. Due to the absence of a gold standard and unknown accuracies, future longitudinal outcome studies are needed to assess the clinical value of 4D-flow in the clinical setting of MVR.

## Background

Mitral valve regurgitation (MVR) is one of the most common valvular heart diseases in western countries and its quantification is challenging due its complex geometry [1]. An accurate assessment of MVR however is crucial for patient risk stratification and optimal decision making towards mitral valve surgery. Furthermore, with the increasing availability of minimally invasive transcatheter treatment options, such as mitral valve transcatheter edge-to-edge repair (TEER), exact quantification of MVR severity and the identification of the underlying mechanism is key for identifying patients who can benefit from less invasive approaches and obviate the need for open heart surgery [2]. Moreover, MVR in hypertrophic cardiomyopathy (HCM) and primary valve disease such as mitral prolapse is still a clinical challenge. In clinical routine, transthoracic and transesophageal echocardiography (TOE) are the primary imaging modalities evaluating MVR and offer the possibility to determine a large number of qualitative (mitral valve leaflet and annular morphology, regurgitant jet size and location) and (semi-) quantitative parameters (vena contracta, regurgitate orifice, fraction and volume) of MVR severity [3]. Nevertheless, the comprehensive echocardiographic evaluation of MVR remains challenging due to the accurate and user dependent positioning of the echo probe, which is prone to bias [3, 4], and Cavalcante et al. [6] and Uretsky et al. [5] have shown in their studies that MVR assessed by cardiac magnetic resonance imaging (CMR) is more reliable than echocardiography in predicting patient outcomes after mitral valve repair.

Four-dimensional (4D) flow CMR is an emerging technology that combines the excellent soft-tissue delineation of conventional CMR with the velocity-encoded quantification of blood flow in three spatial directions [7]. Therefore, in comparison to two-dimensional phase-contrast (2D-PC) CMR, 4D-flow CMR is a potentially more consistent method for flow quantification. 4D-flow can assess blood flow not only across the large vessels but also through cardiac valves and ventricles. Several studies described an association of 4D-flow parameters to hemodynamic characteristics, implicating that 4D-flow is helpful in the evaluation of complex flow conditions such as left ventricular outflow track (LVOT)-obstruction in hypertrophic cardiomyopathy (HCM) [8], atrio-ventricular septal defect repair [9–11], or after valvular heart surgery [12]. Whether 4D-flow might also be used to accurately assess MVR has been evaluated in a few studies [13]. The aim of this systematic review was to identify the potential clinical role of intraventricular 4D-flow in MVR. Furthermore, the reproducibility, technical aspects and comparison against conventional techniques were assessed.

## Methods

Two independent reviewers (i.e., authors YS and BB) conducted a systematic review on the database SCOPUS, MEDLINE and EMBASE database by reading the titles and abstracts [14]. To capture the full spectrum of 4D-flow CMR in MVR quantification, a search matrix with the following combinations of keywords was applied for English original articles, from 2010 until 2021: ((4D) OR (four-dimensional)) AND (flow) AND ((cardiac magnetic resonance imaging) OR (cardiovascular magnetic resonance imaging) OR (magnetic resonance imaging) OR (CMR) OR (MRI)) AND ((mitral valve) OR (left atrioventricular)) AND (regurgitation) OR (insufficiency). Inclusion criteria were the employment of 4D-flow CMR in the evaluation of MVR published in a full-text article until December 2021. The search was done at January 2022. This review was conducted in accordance with the Preferred Reporting Items for Systematic Reviews and Meta-Analysis (PRISMA) statement for reporting systematic reviews [15]. Due to the small number of studies and high heterogeneity in their methodology, a meta-analysis was not conducted.

## Results

**Fig. 1:**
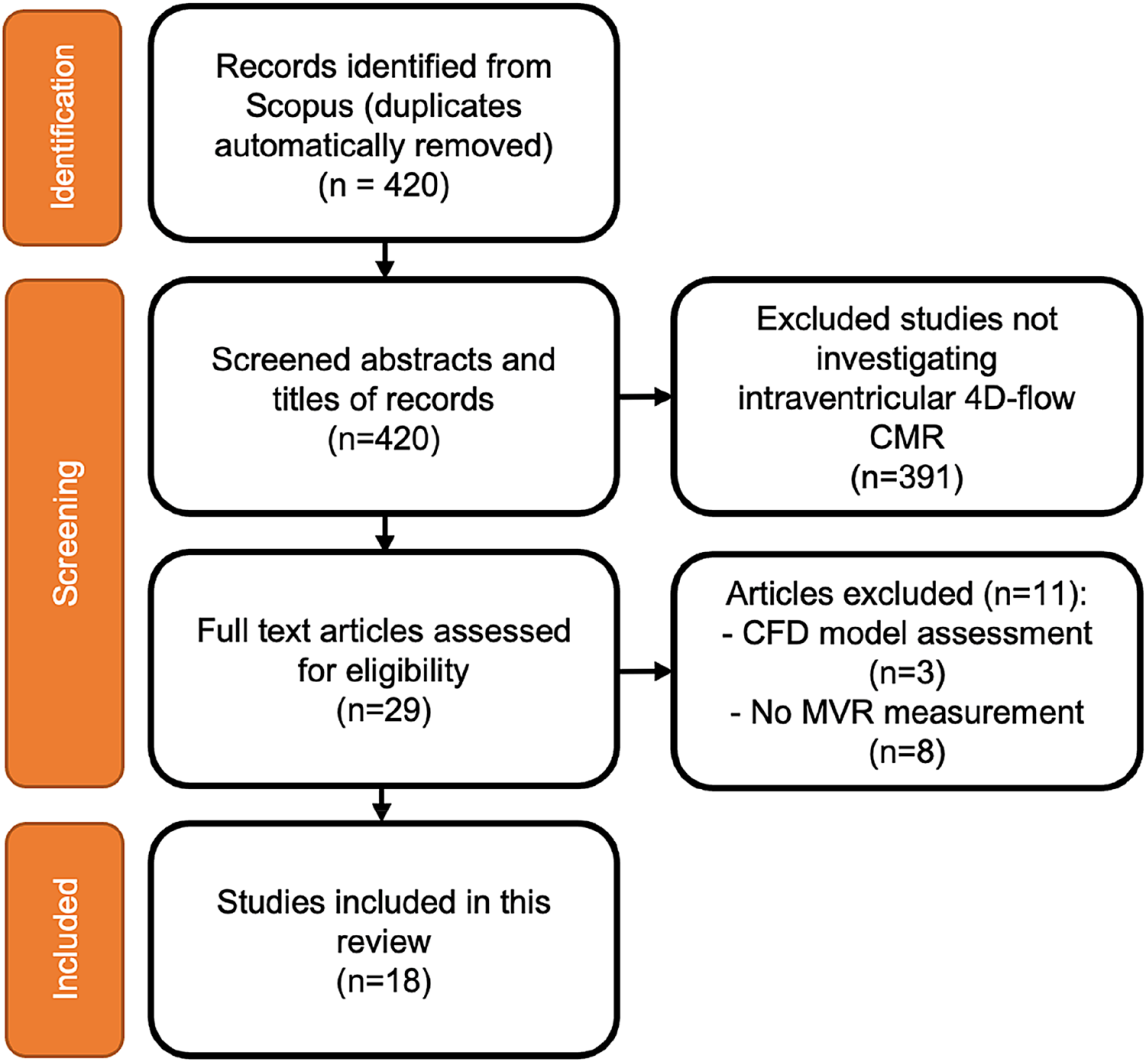
Consort flow of the study selection process. Flow diagram illustrating the stages of the systematic review process in accordance with the Preferred Reporting Items for Systematic Reviews and Meta-Analysis (PRISMA) guidelines [15]. CMR, cardiovascular magnetic resonance imaging; CFD, computational fluid dynamics; MVR, mitral valve regurgitation

The initial search query yielded 420 articles. Based on the mentioned eligibility criteria, 29 articles remained potentially relevant to the current study (Fig. 1). After carefully reviewing the full manuscripts and excluding the studies using computational fluid dynamic (CFD) assessment (n = 3) or not assessing the MVR using 4D-flow methods (n = 8), a total of 18 studies were included in this systematic review, investigating the application of 4D-flow CMR in MVR. Most studies included (n = 12, 67%) were published after 2018, whereas 6 (33%) were studies published in or before 2017.

### Study characteristics and aims

Baseline characteristics of the study cohorts, aim of the studies, publication year, and 4D-flow quantification methods are depicted in Table 1. The main objectives behind these studies were (1) to assess the accuracy and reproducibility of using 4D-flow CMR for quantifying MVR volume (n = 12, 67%), (2) to investigate the association of characteristics of the MVR jet with hemodynamic parameters (n = 3, 17%), and (3) to evaluate LV kinetic energy in patients with underlying cardiac disease and MVR (n = 3, 17%). Additionally, 11 studies (61%) compared patients with underlying cardiac disease and MVR to healthy volunteers for internal validity assessments. Across studies, underlying cardiac diseases such as mitral valve prolapse (MVP) [16], atrial fibrillation (AF) [17], and HCM [18] were included.

**Table 1:** Clinical information summary of the included studies in this systematic review assessing 4D-flow in MVR

First author	Year	Aim of the study	Study type	Population cohort	Mean age (years)	Gender (Male%)	Reproducibility data	MVR evaluation method				
								Echo	2D-PC_Standard_	Volumetric	4D-flow_AIM_	4D-flow_jet_
Fidock et al. [20]	2021	Assess the consistency and reproducibility of various MVR quantification methods using CMR across different etiologies	Prospective	35 patients (unclassified cardiac disease)	66 ± 11	66	✓	✗	✓	✓	✓	✓
Mills et al. [17]	2021	Assess the possibility of obtaining 4D-flow CMR in AF patients and investigate the consistency and reliability of RVT in the assessment of aortic and mitral valvular flow in AF patients versus healthy controls	Prospective	8 AF/10 healthy	62 ± 13/41 ± 20	88/70	✓	✗	✗	✗	✓	✗
Gupta et al. [18]	2021	Evaluate LA KE in HCM patients using 4D-flow CMR and examine coupling correlations with MVR and LVOT obstruction	Retrospective	29 HCM	55.25 ± 9.95	55	✗	✗	✗	✗	✓	✗
Juffermans et al. [25]	2021	Assess interobserver agreement, valvular flow variation, and which variables independently predicted the variance of valvular flow quantification at multiple sites using 4D-flow CMR with automated RVT	Retrospective/ Prospective	64 patients with cardiac disease/76 healthy (20 subjects per site, 7 sites)	32 (24–48)	47	✓	✗	✗	✗	✓	✗
Spampinato et al. [16]	2021	Investigate the clinical efficacy of cine guided valve segmentation of 4D-flow CMR in MVR evaluation in mitral valve prolapse compared to normal routine CMR and TTE	Retrospective	54 mitral valve prolapse/6 healthy	58 ± 14/31 ± 5	78/ 83	✓	✓	✓	✗	✓	✓
Blanken et al. [22]	2020	Assess the accuracy of semiautomated flow tracking against semiautomated RVT in quantifying MVR using 4D-flow CMR data in patients with mild, moderate, or severe MVR	Retrospective	30 MVR	61 ± 10	70	✓	✓	✓	✗	✓	✓
Jacobs K. et al.[19]	2020	Direct evaluation of MVR jets using 4D-flow CMR versus volumetric techniques and as an internal validation approach against annular inflow method	Retrospective	18 CHD with MVR	12.6 ± 7.8	56	✓	✗	✓	✗	✓	✓
Morichi et al. [12]	2020	Determine the effect of annuloplasty in mitral valve repair on LV vortex flow and aortic outflow patterns, and flow energy loss	Prospective	14 MVR/ 20 healthy	64 ± 12/NS	71/ NS	✗	✓	✗	✗	✓	✗
Pruijssen et al. [8]	2020	Evaluate relationships between hemodynamic parameters in HCM patients using 4D-flow CMR	Prospective	13 HCM/11 healthy	51 ± 16/54 ± 15	77/ 73	✓	✗	✗	✗	✓	✗
Kamphuis et al. [26]	2019	Compare 4D-flow CMR with automated RVT to manual RVT in acquired or CHD	Retrospective	114 patients (81 CHD)/46 healthy	17 (13–49)/28 (22–36)	55/ 59	✓	✗	✗	✗	✓	✗
Arvidsson et al. [32]	2018	Investigate hemodynamic forces change in HF patients with LV dyssynchrony using 4D-flow CMR	Retrospective	31 HF and LV dyssynchrony/39 healthy	67 (50–87)/27 (18–63)	77/ 46	✗	✗	✗	✗	✓	✗
Feneis et al. [23]	2018	Determine the consistency and reproducibility of 4D-flow CMR in quantifying MVR in comparison with 2D flow CMR	Retrospective	21 patients	54.1 (21–83)	48	✓	✗	✓	✗	✓	✓
Al-Wakeel et al. [41]	2015	Evaluate LV blood flow dynamics as measured by KE in MVR patients before and after mitral valve repair surgery	Prospective	6 mitral valve repair/4 biological valve replacement/7 healthy	56 ± 9/27 ± 7	70/ NS	✗	✗	✗	✗	✓	✗
Calkoen et al. [21]	2015	Investigate flow patterns in patients with repaired AVSD and healthy controls	Prospective	32 AVSD/30 healthy	25 ± 14/26 ± 12	28/46	✗	✗	✗	✗	✓	✗
Calkoen et al. [11]	2015	Determine the effect of LAVV anomaly on vortex ring generation in AVSD patients	Prospective	32 AVSD/30 healthy	25 ± 14/26 ± 12	28/46	✓	✗	✓	✗	✓	✗
Calkoen et al. [9]	2015	Assess LAVV blood flow and optimize LV inflow quantification in repaired AVSD patients and healthy controls	Prospective	25 AVSD/25 healthy	22 (16–31)/17 [12–28]	28/40	✗	✓	✓	✗	✓	✗
Calkoen et al. [10]	2015	Quantifying LAVV regurgitant jets in corrected AVSD patients using 4D-flow CMR	Prospective	32 AVSD	26 ± 12	28	✗	✓	✓	✗	✓	✗
Hsiao et al. [24]	2015	Evaluate the possibility of measuring net and regurgitant flow volume using 4D-flow CMR across heart valves	Retrospective	34 pediatric CHD	6.9 (0.8–15)	56	✗	✗	✓	✓	✓	✗

The mitral valve regurgitation (MVR) evaluation methods are: (1) echocardiography (Echo), (2) 2D-PC CMR gold standard (2D-PC_Standrad_), (3) volumetric method, (4) 4D-flow_AIM_, and (5) 4D-flow_jet_. CMR, cardiovascular magnetic resonance imaging; AF, atrial fibrillation; RVT, retrospective valve tracking; LA, left atrium; KE, kinetic energy; HCM, hypertrophic cardiomyopathy; LVOT, left ventricular outflow track; TTE, transthoracic echocardiography; CHD, congenital heart disease; HF, heart failure; LV, left ventricle; AVSD, Atrioventricular Septal Defect; LAVV, Left Atrial Ventricular Valve.

### MVR quantification methods

**Fig. 2:**
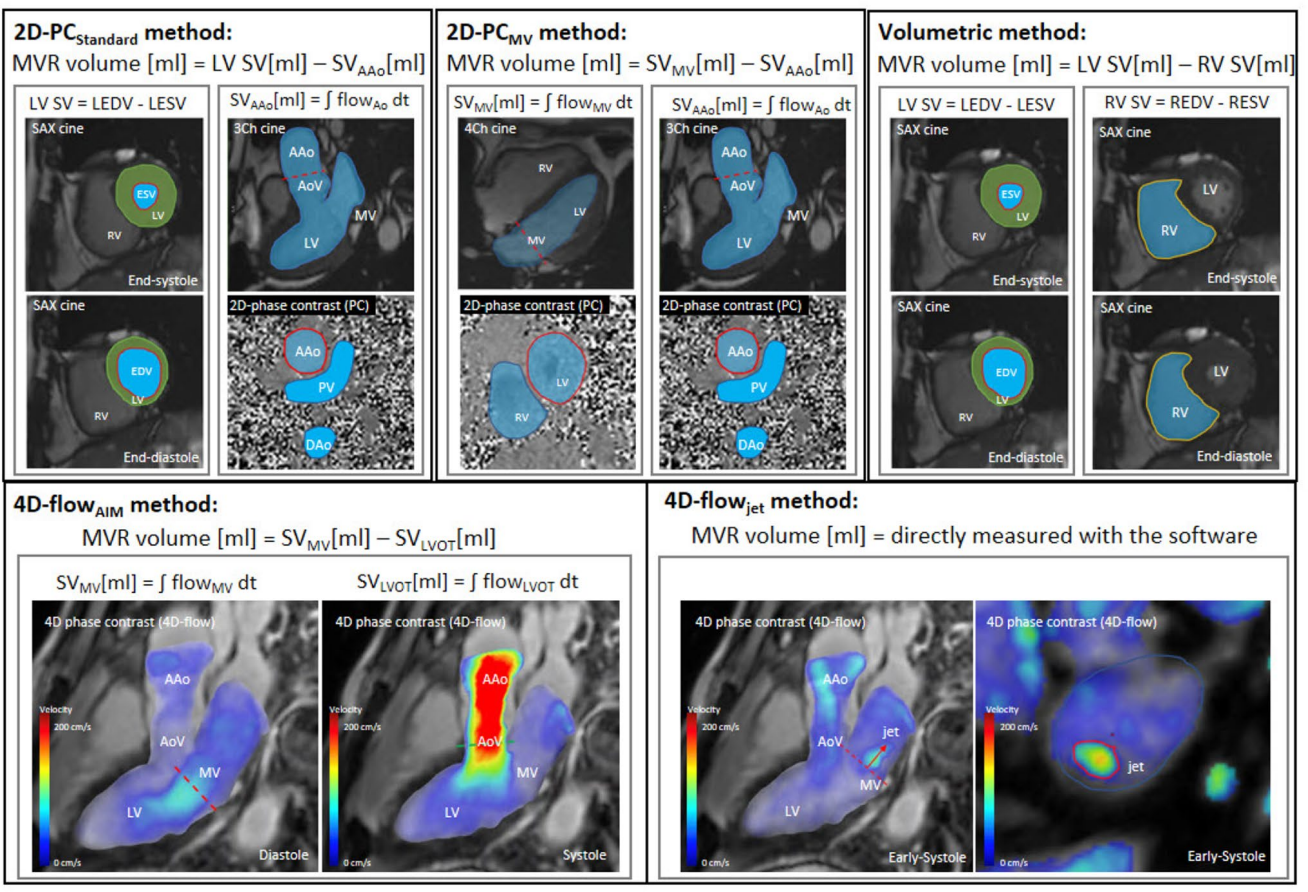
Illustration of MVR quantification methods. 2D-PC_standard_, CMR flow gold standard (Left Ventricle Stroke Volume [LV SV]—Stroke Volume derived from Aortic Forward Flow [SV_AAo_]); 2D-PC_MV,_ directly quantifying flow through Mitral Valve (Stroke Volume derived from Mitral Valve Flow [SV_MV_]—Stroke Volume derived from Aortic Forward Flow [SV_AAo_]); Volumetric (Left Ventricle Stroke Volume [LV SV]—Right Ventricle Stroke Volume [RV SV]); 4D-flow_AIM_ (Stroke Volume derived from Mitral Valve Forward Flow [SV_MV_]—Stroke Volume derived from Aortic Forward Flow [SV_AAo_], or [SV_LVOT_]); 4D-flow_jet_; AoPC, Aortic Forward Flow; EDV, Left Ventricle End Diastolic Volume; ESV, Left Ventricle End Systolic

MVR volume quantification methods require the assessment of stroke volume (SV) either by volumetrically using cine CMR images or by calculation from phase-contrast data. Figure 2 summarizes all the MVR volume quantification methods. (1) The “4D-intraventricular annular inflow method” (4D-flow_AIM_) calculates the regurgitant volume by subtracting the SV derived from aortic forward flow (SV_AAo_) from the SV derived from the forward flow through the mitral valve (SV_MV_), both derived from a single 4D-flow CMR dataset (available in n = 18 studies, 100%). The SV_AAo_ and SV_MV_ are calculated by integrating flows derived from the phase-contrast CMR images over the duration of one cardiac cycle. Additionally, (2) the clinical “2-dimensional phase-contrast standard method” (2D-PC_standard_) is used to indirectly measure the MVR volume by subtracting the SV derived from PC imaging of SV_AAo_ from volumetrically assed LV SV from cine CMR images (n = 8 studies, 44%). The LV SV is calculated by subtracting LV end-diastolic volume (EDV) from LV end-systolic volume (ESV) as derived from short axis cine images of the heart. The remaining methods are (3) “the volumetric method”, which calculates the deviation of the LV SV and right ventricular SV from cine CMR images in 2 (11%) studies, and (4) the 4D-flow_jet_ method directly quantifying the flow and regurgitant volume of the regurgitant jet using 4D-flow CMR in 5 (28%) studies. No study assessed the MVR volume with (5) the “2D-PC mitral valve method” (2D-PC_MV_), which quantifies the MVR volume by subtracting SV_MV_ from LV SV using 2D-PC and cine CMR images, analogous to the 2D-PC_standard_ method. It is important to note, that all quantification approaches, with the exception of the 2D-PC_standard_ method and 4D-flow_jet_ method, require adaptation when significant aortic regurgitation is present. The replacement of the SV of the ascending aorta (AAo) or aortic valve (AoV) by the “net forward flow” through the AAo or AoV (calculated as the SV minus the volume of aortic regurgitation) allows proper quantification of MVR in these cases. Additionally, it is important to note that these methods have limited utility when there is interventricular shunting.

### Technical parameters

Table 2 shows the technical parameters used in the reviewed studies. Scanners magnetic field strengths were 1.5 T (n = 11) and 3 T (n = 11). In all studies, the positioning of the FOV of the 4D-flow sequence was adapted to match a whole heart coverage, especially the entire left-sided cavities and the aortic root. The velocity encoding range (VENC) was set to values around 150 cm/s by default in most studies except in special cases such as congenital heart disease (CHD) [19]. The image resolution was ranging between 0.8 and 4.2 mm^3^, while most studies used a resolution of around 2.5 mm^3^, and a temporal resolution of around 40 ms (21–86 ms). Further acquisition parameters were as follows: echo time (TE) of 1–3 ms, repetition time (TR) of 5–15 ms, the flip angle was mostly 10° (7°–15°), and the mean image acquisition time was generally around 10 min (5–15 min). All studies administrated contrast agents before the 4D-flow acquisition, without specification of the exact timing, and used ECG triggering and respiratory gating. For flow analysis, retrospective valve tracking using post-processing software such as MASS (n = 6) (Leiden University Medical Center, The Netherlands) [9–11, 17, 20, 21] and cvi42 (n = 2) (Circle Cardiovascular Imaging, Calgary, Canada) [8, 18] was common. All studies visually assessed the quality of images and performed pre-processing for de-noising and anti-aliasing.

**Table 2:** Technical parameter summary of the 17 included studies

First author	Vendor	Scanner	Field (T)	VENC (cm/s)	Acquired voxel (mm)	Temporal resolution (ms)	TE (ms)	TR (ms)	Flip Angle (degree)	Cardiac Phase	Acquisition Time (min)
Fidock et al. [20]	Philips	Ingenia/Achieva	1.5, 3	150	3 × 3 × 3	40	3.5	10	10	30	8 ± 4
Mills et al. [17]	Philips	Ingenia	1.5	150	3 × 3 × 3	40	3.5	10	10	30	8–10
Gupta et al. [18]	Siemens	Avanto/Aera /Skyra	1.5, 3	150–250	(2.1–2.8) × (2.1–2.8) × (2.4–3.3)	36.8–40.0	2.2–2.5	4.6–4.9	NaN	NaN	8–15
Spampinato et al. [16]	Philips	Ingenia	1.5	150–250	(0.8–1.47) × (0.8–1.47) × 2.5	38 ± 6	3.3–4.3	7.5–14	10	25	5–10
Blanken et al. [22]	Philips	Intera/Ingenia	1.5	150–280	2.90 × 3.80 × 6.00 / 3.43 × 3.66 × 3.50	21–39	3.3–4.3	8.3–14	10	30	NaN
Jacobs et al. [19]	GE	Optima 450W/MR750	1.5, 3	300 /300	1.2 × 1.4 × 2.1 /0.84 × 0.9 × 1.7	62.5 /32.4	1.9 /1.8	4.4 /4.1	15 /15	20–30	NaN
Morichi et al. [12]	Siemens	Magnetom Skyra	3	150	1.8 × 1.8 × 4.0	67.8	2.86	NaN	8	NaN	< 60
Pruijssen et al. [8]	Siemens	Magnetom Avanto/Aera	1.5	120–250	2.9–4.0 × 2.1–2.8 × 2.8–3.2	37–40	2.2–2.5	4.6–4.9	7–15	NaN	NaN
Kamphuis et al. [26]	Philips	Intera/Ingenia	1.5, 3	150–350	2.3–3.0 × 2.3–3.0 × 3.0	31	3.2	7.7	10	30	5–12
Arvidsson et al. [32]	Philips	Achieva	1.5, 3	NaN	3 × 3 × 3	50	3.1–3.7	5.1–6.3	8	40	28 ± 7
Feneis et al. [23]	GE	MR750	3	400 (250–550)	1.55 × 1.89 × 2.5	53 (37–76)	NaN	NaN	NaN	NaN	11.35 (8.27–14.42)
Al-Wakeel et al. [41]	Philips	Achieva	1.5	150	2.5 × 2.5 × 2.5	NaN	2.6	3.9	5	25	8.5–14
Calkoen et al. [9–11, 21]	Philips	Ingenia	3	150	2.3 × 2.3 × 3–4.2	31	3.2	7.7	10	30	8 (5−12)
Hsiao et al. [24]	GE	TwinSpeed	1.5	150–300	1.04 × 1.38 × 2.41	61 (33–86)	1.8	4.8	15	20	10.17 (7−15)

Multi-center study by Juffermans et al. [25] is excluded due to multiple acquisition parameters presented in the study. VENC, velocity encoding range; TE, echo time; TR, repetition time; NaN, value not indicated in the study.

### Reproducibility and comparison against other methods

Nine studies investigated the MVR quantification reproducibility of 4D-flow_AIM,_ 7 studies (78%) reported good to excellent intra- and inter-reader reproducibility (ICC > 0.8) (Table 3), and the remaining 2 studies described good to excellent intra- but only moderate inter-reader reproducibility [8, 22]. None of the included studies have investigated the inter- and intra-scan reproducibility of 4D-flow acquisition. Seven studies (39%) investigated the agreement of 4D-flow_AIM_ to other MVR acquisition methods [10, 16, 19, 20, 22–24] (Table 4). Inter-modality correlation among the 4 quantification methods was heterogeneous across studies, ranging from moderate to excellent correlation (r > 0.51). In direct comparison to 2D-PC, 4D-flow_AIM_ measurements showed similar intra- and inter-observer agreement [16, 20]. Agreement of these techniques was also associated with the etiology of MVR. In primary MVR, a lower agreement (P > 0.05) was found compared to secondary MVR (P < 0.0001) [20]. When compared to the 2D-PC standard method, 4D-flow_jet_ provided higher MVR volumes (P < 0.05) [20]. Two studies compared 4D-flow_AIM_ to echocardiographic assessment of MVR volumes by the proximal isovelocity surface area (PISA) method with moderate correlation between the two modalities and systematically yielded higher MVR volumes as compared to CMR techniques (mean difference of 15.8 ml) [16].

**Table 3:** Inter- and intra-reader 4D-flow_AIM_ reproducibility data for the included studies in this systematic review

	Intra-reader reproducibility	Inter-reader reproducibility
Fidock et al. [20]	Excellent (CCC = 0.96)	Good (CCC = 0.86–0.96)
Juffermans et al. [25]	N/A	Moderate to Excellent (ICC 0.53–0.97)
Spampinato et al. [16]	Excellent (ICC = 0.98)	Excellent (ICC = 0.92–0.94)
Blanken et al. [22]	N/A	Moderate (r = 0.72)
Jacobs et al. [19]	Excellent (ICC = 0.97–0.98)	Excellent (ICC = 0.94–0.96)
Pruijssen et al. [8]	Good (ICC = 0.83)	Moderate (ICC = 0.73)
Kamphuis et al. [26]	Excellent (ICC = 0.98)	Excellent (ICC = 0.97)
Feneis et al. [23]	Excellent (ICC = 0.98–0.99)	Good to Excellent (ICC = 0.87–0.93)
Calkoen et al. [9]	Good to Excellent (ICC > = 0.77)	Good to Excellent (ICC > = 0.85)

r, sample correlation coefficient; CCC, concordance correlation coefficient; ICC, interclass correlation coefficient; N/A, no value indicated. (r ≥ 0.9, excellent correlation; r  = 0.7–0.89, strong correlation; r = 0.4–0.7, moderate correlation; r  =  0.1–0.39, weak correlation) (ICC ≥ 0.9, excellent correlation; ICC  = 0.75–0.89, good correlation, ICC  =  0.5–0.74, moderate correlation; ICC < 0.5, poor correlation).

**Table 4:** Inter- and intra-modality correlation between 4D-flow_AIM_ and other MVR quantification methods

		4D_AIM_ correlation with			
		2D-PC_Standard_	Volumetric	Echo (PISA)	4D-flow_jet_
Fidock et al. [20]	Inter-modality correlation	Strong (r = 0.82–0.90)	Strong (r = 0.89–0.92)	N/A	Strong (r = 0.85–0.93)
	Intra-Reader Reproducibility	Good (CCC = 0.8)	Good (CCC = 0.88)	N/A	Excellent (CCC = 0.91)
	Inter-Reader Reproducibility	Good (CCC = 0.85–0.95)	Good (CCC = 0.84)	N/A	Moderate (CCC = 0.57–0.60)
Spampinato et al. [16]	Inter-modality correlation	Strong (r = 0.74)	N/A	Moderate (r = 0.63)	Strong (r = 0.76)
Blanken et al. [22]	Inter-modality correlation	Moderate (r = 0.53)	N/A	N/A	N/A
	Inter-Reader Reproducibility	Excellent (r = 0.91)	N/A	N/A	Excellent (r = 0.95)
Jacobs et al. [19]	Inter-modality correlation	Moderate (rho = 0.69–0.70)	N/A	N/A	Strong (rho = 0.80)
	Intra-Reader Reproducibility	Excellent (ICC = 0.97)	N/A	N/A	Excellent (ICC = 0.97)
	Inter-Reader Reproducibility	Excellent (ICC = 0.96)	N/A	N/A	Excellent (ICC = 0.94)
Feneis et al. [23]	Inter-modality correlation	Good to Excellent (ICC = 0.80–0.95)	N/A	N/A	Excellent (ICC = 0.94)
Calkoen et al. [10]	Inter-modality correlation	Moderate (r = 0.65)	N/A	Moderate (rho = 0.51)	N/A
Hsiao et al. [24]	Inter-modality correlation	N/A	Excellent (rho = 0.92)	N/A	N/A

(1) 2D-PC_standard_, (2) volumetric, (3) echocardiography (PISA), and (4) 4D-flow_jet_. r, sample correlation coefficient; CCC, concordance correlation coefficient; rho, population correlation coefficient; ICC, interclass correlation coefficient; N/A, no value indicated. (r  ≥  0.9, excellent correlation; r  = 0.7–0.89, strong correlation; r = 0.4–0.7, moderate correlation; r  =  0.1–0.39, weak correlation) (ICC ≥ 0.9, excellent correlation; ICC  = 0.75–0.89, good correlation, ICC  =  0.5–0.74, moderate correlation; ICC < 0.5, poor correlation).

## Discussion

The findings of the current systematic review on 4D-flow for quantifying MVR volume are as follow: the reviewed studies demonstrated that 4D-flow_AIM_ was the most common used quantification method in the setting of MVR and that the number of articles published are increasing in the recent five years. Moderate to strong agreement between different MVR quantification methods was depicted and reproducibility is generally high, and most authors concluded that 4D-flow_AIM_ has the highest reproducibility across MVR quantification methods. So far, no study linked 4D-flow MVR quantifications to clinical outcomes.

### Comparison of different MVR quantification methods

Due to its widespread availability, simplicity, and affordability, echocardiography by visual assessment and PISA method, remains the most popular modality to evaluate MVR severity. However, echocardiography has some constraints such as variable velocity assessment caused by beam alignment with non-optimal flow convergence, dynamic changes in orifice, limited acoustic window and operator experience. Further, in cases of multiple regurgitant orifices the PISA method is limited. Additionally, when complex flow patterns or complex vessel geometries are present, the calculation of mean velocities and net flow is frequently based on assumptions about the vessel's cross-sectional area or flow profile, which can lead to inaccurate flow quantifications, especially as the regurgitant orifice is not round, but rather oval or irregular in shape [7]. As a result, estimated echo velocity values have a moderate correlation with CMR quantitative measurements. Moreover, among CMR 4D-flow quantification methods might provide additional information with higher reproducibility and robustness in borderline moderate to severe MVR.

2D-PC CMR has become the reference gold standard for clinical aortic forward and backward flow (regurgitation) quantifications because of its high spatial and temporal resolution, simplicity in acquisition and post-processing, and good prognostic and diagnostic outcome data [27]. However, when used for MVR analysis, 2D-PC overestimates the MVR volume by 15% when compared to 4D-flow_AIM_ [28] and is prone to errors because of the two different types of acquisition, 2D-PC and cine images [27]. Besides, concomitant valve disease might impact the accuracy of these measurements. Additionally, the 2D-PC imaging plane should be orthogonal to the flow direction, as stated by Vermes et al. in their study that the misalignment of the 2D-PC imaging plane prevents measuring the aortic peak velocity precisely and reduces the accuracy of flow measurements [29]. The CMR volumetric method based on one cine image acquisition allows a fast and easy assessment of MVR volumes and is a good method for quantifying solitary MVR. However, it is an indirect MVR quantification method, which has poor precision and high segmentation variability for right ventricle SV, and cannot be used in other valves incoherencies [27].

4D-flow CMR acquisitions allow for post-procedural adaptation of the angle and the position of the evaluation planes. 4D-flow has been used frequently for aortic diseases [30, 31], however, using the method in mitral valve disease is more complicated due to the saddle shape and significant through-plane motion of the mitral valve. To directly quantify the regurgitation jet volume with 4D-flow_jet_, proper cine image acquisitions and retrospective valve tracking (RVT) are required. Another advantage of 4D-flow quantification methods is their ability to enable direct valve tracking throughout the cardiac cycle, which is not feasible with 2D-flow imaging due to the motion of the valve annulus. This direct measurement capability is a significant advantage for assessing mitral regurgitation and allows for high reproducibility that might be superior to that of 2D PC methods [13, 23]. Nevertheless, the preferable MVR quantification method by CMR still has to be determined by systematic comparisons of reproducibility and robustness in intra- and inter-reader variability. Moreover, kinetic energy and wall shear stress are some advanced novel 4D-flow intraventricular hemodynamic parameters. For example, Gupta et al. [18] reported that left atrial kinetic energy assessed by 4D-flow is associated with LV obstruction in HCM patients. Whether these novel parameters maybe of advantage and may provide additional information in MVR with a potential clinical impact has to be evaluated in the future. Furthermore, there is no gold-standard MVR grading system by 4D-flow CMR, and the cut-off values are usually decided by the experts at each center. The consensus statement on assessing MVR by CMR suggested a grading system presented in Table 5 [27], however, further studies are required to compare the cut-off values for different quantification methods directly with outcomes.

**Table 5:** Mitral valve regurgitation (MVR) grading system recommended by the consensus statement on assessing MVR by cardiovascular magnetic resonance imaging (CMR). Adapted from consensus Garg et al. [27]

Type of MR	Grading of severity			
	Mild	Moderate	Severe	Very severe
Primary	MR_RF_ < 20%	MR_RF_ = 20–39%	MR_RF_ = 40–50%; MVR > 55–60 ml	MR_RF_ > 50%
Secondary	MVR < 30 ml	MVR = 30–60 ml	MVR > = 60 ml	N/A

MR_RF_, mitral regurgitation fraction

### Limitations of 4D-flow CMR in MVR

Across the reviewed studies, several limitations of 4D-flow CMR require attention, such as long acquisition time [11], using static time-averaged cine images for segmentations [8, 9, 11, 16, 18, 19, 26], difficulties in capturing the exact position of the peak MVR jet [10, 18, 19, 22], low temporal resolution in comparison to other CMR sequences, such as cine bSSFP [8, 20, 32], and the presence of image artifacts in patients with implanted devices [12].

Segmenting 4D-flow images based on time-averaged cine images requires an extra acquisition leading to misalignment between 4D-flow data and the cine images due to heart and patient movements [33]. Unfortunately, the blood-tissue contrast in 4D-flow is very low, which is why an accurate LV segmentation is difficult to perform on the 4D-flow data directly. Current approaches such as in Corrado et al. [34] register automated cine segmentations onto the 4D-flow data for faster analysis. Others, such as in Bustamante et al. [35] use atlas-based segmentations, that means a general segmentation mask is registered onto the 4D-flow CMR data and adapted to the scan. That atlas-based segmentation methods have been used to also train a U-net for direct LV segmentation of cardiac 4D-flow [36]. Prior research has shown that placing the atrioventricular plane at the position of the peak inflow velocity rather than at the height of the valvular plane improves the accuracy of 4D-flow_AIM_ flow velocity estimation [9].

In Garcia et al. [37] a machine learning tool was developed to automatically detect evaluation planes following the mitral valve motion in cine data, which then were interpolated onto 4D-flow data. The need for a measuring plane perpendicular to valvular inflow likely extends to jet planes, which may explain the relatively poor correlation between mitral regurgitation fraction measurements using the volumetric, 4D-flow_jet_, and 4D-flow_AIM_ techniques [19]. Moreover, the limited temporal resolution reduces the overall 4D-flow SNR [32] and affects the velocity profile quality [20] and the measured KE [38].

### 4D-flow acquisition parameters

4D-flow scanning parameters are dependent on many factors, such as the vendor, sequence, and patient’s hemodynamics, as indicated by the 4D-flow consensus statement [7]. The VENC (in cm/s) is often set to be 10% higher than the highest predicted velocity to achieve an acceptable velocity-to-noise ratio (VNR) and avoid aliasing. It is typically about 150 cm/s for MVR quantifications, ranging from 120 to 550 cm/s in the evaluated studies. Aliasing occurs when the VENC value is less than the highest flow velocity, and a high VENC results in a reduced VNR. The FOV of 4D-flow ideally covers the whole heart with the aortic arch. However, it is sufficient to cover the region of interest to decrease scan time, which in the case of MVR quantification is the left ventricle and left atrium. Since the spatial and temporal resolutions impact the accuracy of the flow acquisition, it is best to set them to the highest resolution if there is no time constraint. The temporal resolution is recommended to be lower than 40 ms as stated in the consensus [7], with a range of 21–86 ms. All the reviewed studies used retrospective ECG triggering to cover the whole cardiac cycle and avoid sequence interruptions. However, novel 4D-flow acquisitions use cardiac self-gating techniques [7]. All studies also used respiratory gating to decrease breathing artifacts and scan duration by positioning the navigator on the liver-diaphragm interface. Also, the flip angle varies from 5° to 15°. Overall, it can be concluded that variations in 4D-flow image quality might not be related to technique itself, rather to an inappropriate use of imaging parameters. A consensus of 4D-flow parameters for MVR is still needed.

As opposed to 2D-PC CMR, the 4D-flow analysis uses RVT to quantify eccentric regurgitation jets and correct for annular valve plane motions [10, 13, 26, 28]. In the net forward flow evaluation through cardiac valves, RVT has demonstrated greater accuracy with lesser variance when compared to 2D-PC CMR methods [10, 26, 28]. A multi-center study on assessing the consistency of automated RVT demonstrated that valvular flow measurement can be independent of local CMR scanners and protocols [25].

Even though the optimal setting for MVR quantification remains to be determined, currently used scanners and protocols, still allow for a consistent acquisition of 4D flow sequences [25].

### Outlook on clinical implications

Data on the clinical value of MVR quantification by 4D-flow CMR is scarce and based on small observational studies. To the best of our knowledge, no study exists that links MVR characteristics determined by 4D-flow CMR to the long-term outcome or hard clinical endpoints such as mortality or heart failure events, or remodeling after mitral valve replacement. Conflicting data from large randomized clinical trials on the value of transcatheter mitral valve edge-to-edge repair [39, 40] underline the urgent need for a reproducible and robust quantification of MVR severity that correlates with outcomes and can be used to guide therapeutic decisions [41].

## Limitations

When interpreting the results of this review, it is important to consider several limitations. The results presented show the current role of 4D-flow CMR in the assessment of MVR, which is currently based on descriptive, observational, and primarily retrospective data. The generalizability of our conclusions is reduced by the heterogeneity of the reviewed studies. Without considering factors such as the included study cohorts (healthy controls vs. patients with various cardiac diseases) [10, 12, 22, 32], the severity and mechanism of MVR, and various image acquisition techniques and analysis software packages, and the lack of a gold-standard, it is impossible to compare the values we provided for reproducibility and inter-modality correlation across studies. Further, how the use of contrast agent, the dosage and timing impacts on 4D flow quality is not yet conclusive and needs future evaluation. In addition to the mentioned limitations in the reviewed studies, it is noteworthy to consider the low availability of proper sequences and software in centers and a lack of clinical expertise restricting the broad adoption of clinical 4D-flow CMR [23].

## Conclusions

Intraventricular 4D-flow_AIM_ is the most used 4D-flow method in quantifying MVR among the reviewed studies providing high reproducibility with heterogeneous correlations to conventional quantification methods. Due to the absence of a gold standard, future longitudinal outcome studies need to assess the clinical value of different 4D-flow methods and compare its predictive value to established methods.

## Data Availability

The datasets analyzed during the current study are available via online search using Scopus and Google Scholar.

## Abbreviations

2D-PC: Two-dimensional phase-contrast
AIM: Annular inflow method
CCT: Cardiac computed tomography
CMR: Cardiac magnetic resonance imaging
DICOM: Digital imaging and communications in medicine
HCM: Hypertrophic cardiomyopathy
KE: Kinetic energy
LVOT: Left ventricular outflow track
MVR: Mitral valve regurgitation
PISA: Proximal isovelocity surface area
RVT: Retrospective valve tracking
SV: Stroke volume
TEER: Transcatheter edge-to-edge repair
TOE: Transesophageal echocardiography
VENC: Velocity encoding range
